# Sex Difference in the Association between High-sensitivity C-reactive Protein and Depression: The 2016 Korea National Health and Nutrition Examination Survey

**DOI:** 10.1038/s41598-018-36402-3

**Published:** 2019-02-13

**Authors:** San Lee, Sarah Soyeon Oh, Sung-In Jang, Eun-Cheol Park

**Affiliations:** 1 Department of Public Health, Graduate School, Yonsei University, Seoul, Republic of Korea; 2 Institute of Health Services Research, Yonsei University College of Medicine, Seoul, Republic of Korea; 3 Department of Preventive Medicine, Yonsei University College of Medicine, Seoul, Republic of Korea; 4 Department of Psychiatry and Institute of Behavioral Science in Medicine, Yonsei University College of Medicine, Seoul, Republic of Korea

## Abstract

Elevated levels of circulating high-sensitivity C-reactive protein (hs-CRP) have been observed in depression, with the body mass index (BMI) being a major mediator of this association. However, the sex difference in the association between hs-CRP and depression remains unclear. This study aimed to investigate the sex difference in the association between hs-CRP and depression. Data from the 2016 Korea National Health and Nutritional Examination Survey were used for our study. High hs-CRP was defined as >3.0 mg/L, while depression was determined using a cut-off score of 10 in the Patient Health Questionnaire-9. The study population comprised 5,483 Korean adults. Men with high hs-CRP levels showed statistically higher prevalence of depression than those with low hs-CRP levels (8.90% vs. 3.65%, P < 0.0001). The high hs-CRP group was 1.86 times more likely to have depression after adjusting for BMI and other covariates in men (adjusted odds ratio: 1.86; 95% confidence interval: 1.07–3.25; P = 0.029). Meanwhile, no statistically significant association between hs-CRP and depression was found among women. Depression was considerably associated with hs-CRP only in men, indicating a biological difference between men and women that can independently modify the relationship between hs-CRP and depression.

## Introduction

Increasing evidence supports that depression is associated with inflammatory response^1,2^. Systemic immune activation, i.e., increased levels of pro-inflammatory cytokines and changes in the acute-phase protein response, has been reported in major depression^3,4^. Therefore, depression may be viewed as a psychoneuroimmunological disorder that shows persistent inflammation^3^.

C-reactive protein (CRP) is a positive acute response protein that is related to systemic inflammation^5^. Elevated levels of circulating CRP have been observed in depression^6–9^, but the exact association between high CRP and depression is unclear^10–12^. Although CRP levels are generally only elevated in severe inflammation, the development of high-sensitivity assays (e.g., hs-CRP test) allowed for the quantification of low CRP levels in healthy individuals. Thus, studies on the clinical role of hs-CRP in the association between low-grade systemic inflammation and depression are expected to be undertaken.

Several studies have investigated the association between depression and CRP according to sex, but the results are conflicting. A 1-year observation showed that major depression was strongly associated with increased levels of CRP in men^13^. In a cohort study conducted in northern Finland, although the association of depression with hs-CRP was not considerable in women, elevated hs-CRP levels (≥1.0 mg/L) increased the probability for severe current and recurrent depressive episodes in men by 1.7-fold and 3.1-fold, respectively^14^. A study using 6-year data from the National Health and Nutrition Examination Survey (NHANES) also showed that CRP remained profoundly associated with depression in a dose-response fashion in men, but not in women^15^. By contrast, a retrospective cohort study of 1,494 female patients reported that hs-CRP is an independent risk factor for de novo major depressive disorder in women^8^. In a study using combined data of two Copenhagen population-based studies of 73,131 samples, elevated CRP levels were associated with increased risk for depression after adjusting for covariates including sex^9^.

The body mass index (BMI, kg/m^2^) has been reported to be a major mediator of the inflammation-depression association^4^. In an epidemiological study, CRP was associated with depression only in obese men^16^. A study of 13,006 adults from the NHANES showed no association between CRP and depression after adjusting for BMI^15^. Moreover, abnormal body weight, both under- and overweight, has been suggested to profoundly influence the relationship between CRP and depression. In the US general population, higher BMI was reported to be associated with higher CRP concentrations^17^. Given that the adipose tissue of people with obesity secretes higher amounts of inflammatory molecules including CRP^18,19^, it is possible that the BMI-CRP association is at least in part related with depression.

Given these results, we aimed to investigate the probability that depression differed in men and women with different hs-CRP levels. In addition, we also aimed to evaluate the effect of the association between hs-CRP and depression after adjusting for covariates including BMI in the Korean general population, which comprise individuals with relatively lower BMI than that of the Western population.

## Results

### Descriptive statistics

The study comprised 5,483 participants; of these, 2,373 were men and 3,110 were women. The general characteristics of the study population by sex are shown in Table 1. A total of 4.17% and 7.52% male and female participants had depression, respectively. Men with high hs-CRP levels showed a statistically higher prevalence of depression compared to men with low hs-CRP levels (8.90% vs. 3.65%, P < 0.0001). Meanwhile, the prevalence of depression in women was not significantly different between the groups of high and low hs-CRP levels (9.79% vs. 7.34%, P = 0.215).

**Table 1:** Sociodemographic characteristics of the study participants according to the presence and absence of depression (PHQ-9 ≥ 10).

	Men			Women		
	Depressive	Nondepressive	*p*-value	Depressive	Nondepressive	*p*-value
**Serum hs-CRP**			**<0.0001**			0.215
Low: less than or equal to 3.0 mg/L	78 (3.65)	2,059 (96.35)		211 (7.34)	2,664 (92.66)	
High: above 3.0 mg/L	21 (8.90)	215 (91.10)		23 (9.79)	212 (90.21)	
**Age (years)**			0.678			**<0.0001**
20–29	11 (4.37)	241 (95.63)		29 (8.66)	306 (91.34)	
30–39	20 (4.67)	408 (95.33)		38 (6.61)	537 (93.39)	
40–49	16 (3.43)	450 (96.57)		20 (3.39)	570 (96.61)	
50–59	12 (2.86)	407 (97.14)		37 (6.17)	563 (93.83)	
60–69	21 (5.02)	397 (94.98)		49 (9.53)	465 (90.47)	
70–79	15 (4.78)	299 (95.22)		52 (13.58)	331 (86.42)	
≥80	4 (5.26)	72 (94.74)		9 (7.96)	104 (92.04)	
**Educational attainment**			**<0.0001**			**<0.0001**
Elementary school and below	31 (8.68)	326 (91.32)		101 (43.16)	685 (23.82)	
Middle school	10 (4.05)	237 (95.95)		33 (14.10)	289 (10.05)	
High school	30 (3.87)	746 (96.13)		55 (23.50)	902 (31.36)	
University or above	28 (2.82)	965 (97.18)		45 (19.24)	1,000 (34.77)	
**Equalized household income**			**<0.0001**			**<0.0001**
Quartile 1 (low)	43 (43.43)	363 (15.96)		98 (12.85)	517 (87.15)	
Quartile 2	28 (28.28)	557 (24.49)		58 (10.25)	710 (89.75)	
Quartile 3	16 (16.17)	658 (28.94)		56 (5.75)	793 (94.25)	
Quartile 4 (high)	12 (12.12)	696 (30.61)		22 (4.31)	856 (95.69)	
**Marital status**			**<0.0001**			**<0.0001**
Married	47 (2.63)	1,743 (97.37)		118 (5.49)	2,033 (94.51)	
Separated/divorced/widowed	23 (15.03)	130 (84.97)		82 (14.34)	490 (85.66)	
Never married	29 (6.74)	401 (93.26)		34 (8.79)	353 (91.21)	
**Alcohol use status**			0.822			0.347
No	27 (3.95)	656 (96.05)		145 (7.92)	1,685 (92.08)	
Yes	72 (4.26)	1,618 (95.74)		89 (6.95)	1,191 (93.05)	
**Smoking status**			**<0.0001**			**<0.0001**
Non-smoker	47 (2.91)	1,568 (97.09)		203 (6.79)	2,788 (93.21)	
Smoker	51 (6.86)	706 (93.14)		31 (26.05)	88 (73.95)	
**Chronic medical disease**			**0.002**			**<0.0001**
None	48 (3.25)	1,431 (96.75)		107 (5.25)	1,930 (94.75)	
One	21 (4.38)	459 (95.63)		57 (10.16)	504 (89.84)	
Two or more	30 (7.25)	384 (92.75)		70 (13.67)	442 (86.33)	
**Residential area**			0.313			0.249
Urban	34 (3.66)	896 (96.34)		87 (6.87)	1,180 (93.13)	
Rural	65 (4.50)	1,378 (95.50)		147 (7.98)	1,696 (92.02)	
**BMI**			**0.026**			**0.019**
Underweight	7 (11.67)	53 (88.33)		7 (4.90)	136 (95.10)	
Normal weight	56 (4.16)	1291 (95.84)		133 (6.67)	1860 (93.33)	
Overweight	30 (3.58)	809 (96.42)		77 (9.53)	731 (90.47)	
Obesity	6 (4.72)	121 (95.28)		17 (10.24)	149 (89.76)	
**Menopause (females only)**						**<0.0001**
No				85 (5.51)	1459 (94.49)	
Yes				149 (9.51)	1417(90.49)	
**Participants**	99 (4.17)	2274 (95.83)		234 (7.52)	2876 (92.48)	

Categorical variables are presented as numbers and percentages.

PHQ-9, patient health questionnaire-9; hs-CRP, high-sensitivity C-reactive protein; BMI, body mass index.

In general, men with depression tended to have low educational level, low house income, current unmarried status, be smoking, have more chronic medical disease, and low BMI. Meanwhile, no statistical difference for age, alcohol use, and residential area by prevalence of depression was noted among men. For women participants, all variables reached considerable difference according to prevalence of depression, except hs-CRP, alcohol use status, and residential area.

### Sex difference of the association between hs-CRP and depression

Table 2 shows the results of multivariate logistic regression analysis for the association of depression with hs-CRP. In men, the high hs-CRP group was 1.86 times more likely to have depression after adjusting for covariates (adjusted Odds Ratio (OR): 1.86; 95% Confidence Interval (CI): 1.07–3.25; P = 0.029). Low house income, unmarried status, smoking, and two or more chronic medical diseases were found to considerably increase the probability of depression in men. Meanwhile, age, educational level, alcohol use, residential area, and BMI showed no substantial difference in the prevalence of depression in men.

**Table 2:** Results of the multivariate logistic regression analysis for the association between hs-CRP and depression (PHQ-9 ≥ 10).

	Depression (PHQ-9 ≥ 10)							
	Men				Women			
	OR	95% CI		*p*-value	OR	95% CI		*p*-value
Serum hs-CRP								
Low: less than or equal to 3.0 mg/L	1.00				1.00			
High: above 3.0 mg/L	**1.86**	**1.07**	**3.25**	**0.029**	1.10	0.67	1.80	0.704
Age (years)								
20–29	1.00				1.00			
30–39	1.90	0.80	4.50	0.146	0.93	0.47	1.85	0.840
40–49	1.34	0.52	3.44	0.547	**0.33**	**0.15**	**0.74**	**0.007**
50–59	0.60	0.20	1.84	0.373	**0.19**	**0.06**	**0.60**	**0.005**
60–69	0.75	0.24	2.41	0.633	**0.15**	**0.04**	**0.50**	**0.002**
70–79	0.45	0.13	1.55	0.205	**0.13**	**0.04**	**0.46**	**0.001**
≥80	0.40	0.08	1.92	0.250	**0.06**	**0.01**	**0.24**	**<0.0001**
Educational attainment								
Elementary school and below	1.00				1.00			
Middle school	0.51	0.23	1.15	0.105	0.90	0.57	1.43	0.654
High school	0.51	0.26	1.01	0.055	**0.50**	**0.30**	**0.83**	**0.007**
University or above	0.48	0.23	1.02	0.055	**0.46**	**0.26**	**0.83**	**0.009**
Equalized household income								
Quartile 1 (low)	1.00				1.00			
Quartile 2	**0.47**	**0.27**	**0.83**	**0.009**	**0.52**	**0.36**	**0.77**	**0.001**
Quartile 3	**0.26**	**0.13**	**0.51**	**<0.0001**	**0.54**	**0.36**	**0.82**	**0.004**
Quartile 4 (high)	**0.20**	**0.09**	**0.41**	**<0.0001**	**0.23**	**0.13**	**0.40**	**<0.0001**
Marital status								
Married	1.00				1.00			
Separated/divorced/widowed	**3.55**	**1.96**	**6.41**	**<0.0001**	**1.70**	**1.19**	**2.44**	**0.003**
Never married	**2.52**	**1.33**	**4.79**	**0.005**	1.51	0.81	2.84	0.196
Alcohol use status								
No	1.00				1.00			
Yes	1.21	0.74	1.98	0.460	0.94	0.69	1.28	0.673
Smoking status								
Non-smoker	1.00				1.00			
Smoker	**2.16**	**1.38**	**3.38**	**0.001**	**4.04**	**2.51**	**6.49**	**<0.0001**
Chronic medical disease								
None	1.00				1.00			
One	1.68	0.90	3.12	0.104	**2.04**	**1.34**	**3.10**	**0.001**
Two or more	**2.78**	**1.50**	**5.18**	**0.001**	**2.45**	**1.58**	**3.80**	**<0.0001**
Residential area								
Urban	1.00				1.00			
Rural	1.07	0.68	1.68	0.773	1.12	0.84	1.50	0.437
BMI								
Underweight	1.93	0.75	4.97	0.170	0.72	0.32	1.62	0.429
Normal weight	1.00				1.00			
Overweight	0.82	0.51	1.33	0.427	1.08	0.79	1.49	0.628
Obesity	0.73	0.29	1.82	0.494	0.90	0.50	1.63	0.735
Menopause (females only)								
No					1.00			
Yes					2.12	0.84	5.34	0.110

PHQ-9, patient health questionnaire-9; hs-CRP, high-sensitivity C-reactive protein; BMI, body mass index; OR, odds ratio; CI, confidence interval.

There were no significant associations between hs-CRP levels and depression in the women participants (adjusted OR: 1.10; 95% CI: 0.67–1.80; P = 0.704). Age was found to have inverse correlation with the prevalence of depression in women. Low educational level, low house income, smoking, and chronic medical conditions were associated with increased probability of depression in women. Meanwhile, alcohol use, residential area, BMI, and menopausal status was not associated with depression in women. The detailed results of multivariate logistic regression are shown in Table 2.

When we performed multiple linear regression analysis using hs-CRP and PHQ-9 score for depression as continuous variables, the association between the two variables remained significant in men (β = 0.067, standard error (SE) = 0.026, P = 0.009) (See Supplementary Table S1). However, there were no significant association between hs-CRP and depression in women (β = 0.041, SE = 0.033, P = 0.223), and this showed the same pattern of association by sex as in the results of multiple logistic regression analysis.

### Combined effect of hs-CRP and each covariate on depression

The results of subgroup analysis showing the combined effects of hs-CRP and sociodemographic variables on the prevalence of depression are presented in Tables 3 and 4. The association between BMI and hs-CRP in depression was only found in the “normal weight” group in men. The “normal weight” group with high hs-CRP levels were 2.46 times more likely to have depression than those with low hs-CRP levels.

**Table 3:** Subgroup analysis of the association between hs-CRP and depression (PHQ-9 ≥ 10) stratified by sociodemographic variables in men.

Men	Low hs-CRP (≤3.0 mg/L)		High hs-CRP (>3.0 mg/L)			
	OR		OR	95% CI		*p*-value
Age (years)						
20–29	1.00	**5.65**		**1.36**	**23.52**	**0.017**
30–39	1.00	0.50		0.07	3.82	0.503
40–49	1.00	0.97		0.12	7.59	0.976
50–59	1.00	**9.18**		**2.83**	**29.79**	**<0.0001**
60–69	1.00	**3.47**		**1.23**	**9.45**	**0.015**
70–79	1.00	1.77		0.48	6.57	0.393
≥80	1.00	1.38		0.13	14.30	0.786
Educational attainment						
Elementary school and below	1.00	2.35		0.99	5.60	0.053
Middle school	1.00	3.33		0.81	13.66	0.094
High school	1.00	**3.30**		**1.36**	**7.99**	**0.008**
University or above	1.00	1.28		0.38	4.31	0.695
Equalized household income						
Quartile 1 (low)	1.00	1.96		0.95	4.02	0.069
Quartile 2	1.00	1.95		0.71	5.32	0.195
Quartile 3	1.00	1.78		0.39	8.04	0.456
Quartile 4 (high)	1.00	2.58		0.55	12.12	0.229
Marital status						
Married	1.00	1.46		0.61	3.49	0.397
Separated/divorced/widowed	1.00	**3.12**		**1.16**	**8.36**	**0.024**
Never married	1.00	**3.13**		**1.25**	**7.82**	**0.015**
Alcohol use status						
No	1.00	2.18		0.69	6.86	0.184
Yes	1.00	**2.68**		**1.53**	**4.68**	**0.001**
Smoking status						
Non-smoker	1.00	**2.29**		**1.09**	**4.83**	**0.030**
Smoker	1.00	**2.82**		**1.41**	**5.62**	**0.003**
Chronic medical disease						
None	1.00	**2.29**		**1.05**	**5.01**	**0.038**
One	1.00	2.45		0.86	6.95	0.093
Two or more	1.00	**2.55**		**1.07**	**6.04**	**0.034**
Residential area						
Urban	1.00	**2.68**		**1.13**	**6.35**	**0.025**
Rural	1.00	**2.51**		**1.35**	**4.65**	**0.004**
BMI						
Underweight	1.00	3.84		0.59	25.19	0.161
Normal weight	1.00	**2.46**		**1.20**	**5.01**	**0.014**
Overweight	1.00	2.28		0.91	5.74	0.081
Obesity	1.00	3.84		0.73	20.19	0.112

PHQ-9, patient health questionnaire-9; hs-CRP, high-sensitivity C-reactive protein; BMI, body mass index; OR, odds ratio; CI, confidence interval.

**Table 4:** Subgroup analysis of the association between hs-CRP and depression (PHQ-9 ≥ 10) stratified by sociodemographic variables in women.

Women	Low hs-CRP (≤3.0 mg/L)	High hs-CRP (>3.0 mg/L)			
	OR	OR	95% CI		*p*-value
Age (years)					
20–29	1.00	1.34	0.29	6.15	0.704
30–39	1.00	1.39	0.47	4.10	0.554
40–49	1.00	**4.50**	**1.42**	**14.29**	**0.011**
50–59	1.00	1.26	0.37	4.28	0.717
60–69	1.00	0.48	0.11	2.05	0.320
70–79	1.00	1.04	0.42	2.59	0.938
≥80	1.00	2.19	0.41	11.79	0.361
Educational attainment					
Elementary school and below	1.00	0.98	0.49	1.97	0.957
Middle school	1.00	2.86	0.98	8.33	0.055
High school	1.00	1.36	0.52	3.52	0.532
University or above	1.00	1.05	0.32	3.46	0.942
Equalized household income					
Quartile 1 (low)	1.00	1.06	0.53	2.11	0.864
Quartile 2	1.00	1.08	0.42	2.81	0.874
Quartile 3	1.00	2.12	0.92	4.93	0.080
Quartile 4 (high)	1.00	<0.001	<0.001	>999.99	0.973
Marital status					
Married	1.00	1.44	0.78	2.68	0.247
Separated/divorced/widowed	1.00	0.92	0.40	2.12	0.850
Never married	1.00	2.34	0.75	7.34	0.143
Alcohol use status					
No	1.00	1.47	0.77	2.80	0.238
Yes	1.00	1.24	0.65	2.36	0.515
Smoking status					
Non-smoker	1.00	1.00	0.58	1.72	0.985
Smoker	1.00	**5.77**	**1.72**	**19.34**	**0.005**
Chronic medical disease					
None	1.00	1.48	0.75	2.90	0.259
One	1.00	2.01	0.92	4.37	0.078
Two or more	1.00	0.56	0.20	1.62	0.286
Residential area					
Urban	1.00	1.43	0.70	2.95	0.331
Rural	1.00	1.34	0.75	2.39	0.329
BMI					
Underweight	1.00	<0.001	<0.001	>999.99	0.981
Normal weight	1.00	1.12	0.53	2.34	0.775
Overweight	1.00	1.10	0.51	2.37	0.818
Obesity	1.00	2.28	0.81	6.43	0.120
Menopause (females only)					
No	1.00	1.64	0.77	3.50	0.203
Yes	1.00	1.16	0.66	2.03	0.612

PHQ-9, patient health questionnaire-9; hs-CRP, high-sensitivity C-reactive protein; BMI, body mass index; OR, odds ratio; CI, confidence interval.

The combined effect of smoking and hs-CRP on depression differed between men and women. The association was linear in men and threshold-effect in women, with only current smoking showing a substantial association with depression (OR: 5.77; 95% CI: 1.72–19.34). Residential area and hs-CRP were linearly associated with depression among men (OR: 2.68 and 2.51; 95% CI: 1.13–6.35 and 1.35–4.65, respectively), but the association was not profound in women. Compared to current married status, unmarried status showed considerable combined effect with hs-CRP on depression only in men (OR: 3.12 and 3.13; 95% CI: 1.16–8.36 and 1.25–7.82, respectively). Current alcohol use and hs-CRP were associated with depression in a threshold effect only in men (OR: 2.68; 95% CI: 1.53–4.68). The association of hs-CRP with each age or educational attainment category did not show a considerable trend in combined effect to depression in both men and women. House income in both sex, and menopausal status in women was not associated with hs-CRP in depression. The results of subgroup analyses are presented in detail in Tables 3 and 4.

In addition, we performed subgroup analysis on men stratified by chronic medical diseases, which was considerably associated with depression in men (See Supplementary Table S2). In multivariate logistic regression analysis, the association between hs-CRP and depression was significant in men without or with one chronic medical disease (adjusted OR 2.12; 95% CI 1.06–4.24; P = 0.033). There was no association between hs-CRP and depression in men with two or more chronic medical diseases (adjusted OR 1.46; 95% CI 0.53–4.01; P = 0.462).

## Discussion

Using hs-CRP categories recommended to assess the high risk for cardiovascular disease^20^, we found that depression was associated with high hs-CRP levels in men. After adjusting for potential confounding covariates, elevated hs-CRP (>3.0 mg/L) increased the prevalence of depression independently in men by nearly twofold. Moreover, the association between hs-CRP and depression in multiple linear regression analysis remained significant in men only.

Most literatures have revealed elevated inflammatory markers in patients with mood disorders such as depression^21–23^. A growing body of evidence suggests that diseases with immune activation and increased CRP levels predict increased risk of depression^9,21,24^. It is also suggested that, in addition to inflammatory conditions and neuro-inflammatory disorders, obesity^25^ and life stressors^26^ may also involve inflammation process and induce clinical depression. Depression itself can also trigger subsequent CRP elevation^27,28^. Therefore, it can be said that depression and inflammation are intertwined, fueling and feeding off each other^29^. Although the underlying bidirectional mechanisms are still poorly understood, our findings consistently show this association between depression and inflammation. The results of our study also support that of earlier epidemiological studies^6,13,14,30,31^.

Meanwhile, in accordance with previous studies^16,32^, we found an association between depression and elevated hs-CRP levels in men only. Several studies reported significantly elevated levels of pro-inflammatory cytokines, including interleukin-6 (IL-6), in men compared to women during sepsis^33–35^. Since CRP is produced by hepatocytes largely under regulatory control of inflammatory cytokines, including IL-6^36^, the biological difference of pro-inflammatory cytokines during inflammatory conditions by sex might be reflected in our results. Differential effects of sex hormones may also explain this sex difference. A review article of studies in sepsis showed that female sex hormones exhibit protective effects, whereas male sex hormones can be suppressive on cell-mediated immune responses^37^.

A previous study^14^ reported an association between hs-CRP and depression in elderly women but not in young participants and suggested that this could be explained by hormonal changes during the aging process. Hormonal changes related to menstrual cycle was also suggested as a possible modulator between hs-CRP and depression^13^. By contrast, our subgroup analysis showed that the association of hs-CRP with menopausal status in women had no substantial effect on depression and depression was not related with hs-CRP in elderly women in our multivariate logistic analysis. For now, considering the possible uncontrolled confounding effects of oestrogen treatment and other hormonal changes in women, we cannot present any additional input regarding this debate. Body image perception^38,39^ and CRP-related genetic variation by sex could be also considered as other possible explanations of such discrepancy among studies^40,41^. Further research to examine the sex difference in the association between hs-CRP and depression focusing on modulating effects of hormonal change, body image, genetic variation, and other possible related factors, is needed.

A study conducted in Germany reported that depression is considerably associated with CRP in obese men but not in non-obese men^14^. By contrast, a study comprising 6,901 Chinese participants showed that depression was profoundly negatively associated with BMI, with underweight being associated with worse depressive symptoms than other BMI groups^42^. In our results, hs-CRP was considerably associated with depression in men after adjusting BMI. These inconsistencies could be related to the differences in sample characteristics, such as the number of participants in the BMI categories, age groups, and ethnicity. The Korean general population analysed in this study primarily comprised individuals with relatively low BMI than those in other previous studies conducted in Europe and North America. This difference among studies might confound the effect of BMI on the association between hs-CRP and depression.

In this study, a cut-off value of 3 for hs-CRP, which was originally defined in non-psychiatric studies, was used to categorize the participants. A previous study revealed that more than one-third of schizophrenia, unipolar depression, bipolar depression, and bipolar mania had CRP level >3 mg/L, and suggested that it might be related to increased risk of cardiovascular events in those patients^43^. Moreover, several other studies in the psychiatric field used the same cut-off to evaluate the association between hs-CRP and depression^44^, antidepressant consumption^9,45^, reduced quality of life^46^, and mortality^47^ in psychiatric patients. Following the cut-off of hs-CRP in these studies, we presented our data with the same cut-off value.

The strengths of our study were that our data were obtained from a genetically homogeneous Korean adult population. This homogeneity is noteworthy because CRP levels are known to differ substantially between races^48,49^. All the participants of the study were Asians. Moreover, the BMI in our study population was relatively lower than that in several previous studies^6,16,50^, which could shed light on the association between hs-CRP and depression in the population with relatively low BMI. In addition, the scale used to define depression (PHQ-9) has been shown to be a valid scale for screening depression in the general population^51^.

There were also several limitations to our study. First, this study was conducted using cross-sectional association data that are unable to clarify whether systemic inflammation precedes the onset of depression or occurs as a part of the somatic presentations of the depression. Second, there may be unrecognized confounding factors as in all observational studies. Such confounding factors could have contributed to the inconsistent association between hs-CRP and depression. For instance, acute infection^52^ and seasonal variation^53,54^ might impact CRP measures, and oestrogen therapy has been considered as a factor associated with hs-CRP levels among women^55^. However, relevant data were not collected in the 2016 KNHANES and thus were not included as variables in our analyses. Third, KNHANES was designed to collect data from non-institutionalized population only, and these data may represent only less severe depression because those who are severely depressed may be institutionalized or disproportionately chosen not to participate in the survey. This could limit the statistical power of the analysis by not reflecting the severity of depression. In addition, we did not include information on prior diagnosis for depression and treatment experience for the disorder, and this could confound the comparison among groups. Lastly, the lack of information on medications, particularly psychotropics, could attenuate the real association between hs-CRP and depression and might affect the results.

Despite these limitations, this study provides additional evidence that sex plays a critical role in the relationship between hs-CRP and depression. In addition, the relatively low BMI of the study population emphasizes the independent association of hs-CRP with depression after controlling for the effect of BMI, which is considered to be a major mediator of the inflammation-depression relationship. Our findings support the depression-inflammation relationship in the Asian population.

## Conclusions

In conclusion, depression was considerably associated with hs-CRP only in men, even after adjusting for age, BMI, and other variables known to affect low-level inflammation. This finding suggests that a biological difference between men and women that can independently modify the relationship between hs-CRP and depression. Future investigations are needed to elucidate the possible association between systemic inflammation and depression by sex.

## Methods

### Study population and data

This study was conducted using data from the 2016 Korea National Health and Nutrition Examination Survey (KNHANES). The KNHANES is a nationwide population-based survey of the health and nutritional status of Koreans conducted by the Korea Centers for Disease Control and Prevention. The survey aims to evaluate the health and nutritional status of South Koreans and provide data for the development and evaluation of health policies and programs in Korea. The survey also produces statistical data regarding smoking, drinking, physical activities, and obesity for the World Health Organization and the Organization for Economic Cooperation and Development.

The KNHANES is annually conducted in twenty households throughout 192 regions, and 10,000 individuals aged ≥1 year are targeted for the survey. The survey pool is divided into three groups according to age: children (1–11 years), adolescents (12–18 years), and adults (≥19 years), and are categorized according to the different survey components. Adult participants completed the health interview survey that included the Patient Health Questionnaire-9 (PHQ-9), a depression screening scale. In this study, we included respondents aged ≥20 years who participated in the 2016 KNHANES, responded to the PHQ-9 questions, and had available results on hs-CRP levels. All subjects who participated in the survey signed an informed consent form. The 2016 KNHANES complied with the tenets of the Declaration of Helsinki and the survey was exempted from IRB review, according to government regulation. Ethical approval was not required for this study as 2016 KNHANES provides secondary data that is publicly available, de-identified data.

### Measures

#### Patient Health Questionnaire-9 (PHQ-9)

The Patient Health Questionnaire (PHQ)^56^ is a depression screening module that is a component of the Primary Care Evaluation of Mental Disorders instrument^57^. The PHQ-9 is a nine-item self-administered version of the PHQ and has been validated as a reliable depression screening tool and a measure of depression severity^51^. It is based on the Diagnostic and Statistical Manual of Mental Disorders, Fourth Edition criteria for major depression^58^. The brevity and face validity of the PHQ-9 have made it a popular choice as a screening instrument of depression. Each item on the PHQ-9 is scored on a scale of 0 to 3, after which the scores are added to yield a total score ranging between 0 and 27. In its initial validation study, a score of 10 or higher had a sensitivity of 88% and a specificity of 88% for detecting major depressive disorders^51^. The Korean version of the PHQ-9 also set the cut-off value at 10 to achieve a sensitivity of 81.8% and a specificity of 89.9% for identifying major depressive disorders^59^. In the current study, depression was defined as a PHQ-9 of ≥10.

#### Hs-CRP

Serum hs-CRP levels were measured via the Roche immunoturbidimetric ‘CRP’ and ‘C-reactive protein (latex) high sensitivity’ methods. Specimens (0.1–20 mg/L) were analysed using the high-sensitivity assay. Serum samples used for the analysis of hs-CRP were stored at 2–8 °C in refrigerated containers after blood test was taken. All laboratory analyses were performed within 24 hours of sample collection. To compare the prevalence of depression among different levels of hs-CRP, hs-CRP level of 3.0 mg/L was used. High hs-CRP levels were defined as hs-CRP levels >3.0 mg/L, which was the same as the cut-off stipulated by the American Heart Association and Centers for Disease Control and Prevention to indicate “high risk” for cardiovascular disorder^20^. Meanwhile, low hs-CRP levels were defined as <3.0 mg/L.

#### Covariates

Demographic (age and residential area), socioeconomic (educational attainment, household income, and marital status), and health-related (BMI, alcohol use status, smoking status, chronic medical diseases, and menopausal status in women) covariates were included in this study. Chronic medical diseases that were reported to be directly or indirectly associated with hs-CRP levels, namely, hypertension^60^, diabetes mellitus^61^, dyslipidaemia^62^, coronary heart disease including myocardial infarction and angina pectoris^63^, stroke^64^, and rheumatoid arthritis^65^, were included as medical comorbidities.

#### Statistical analysis

Chi-square test was used to evaluate and compare the general characteristics of the study participants. Multivariate logistic regression analysis and multivariate linear regression analysis were used to examine the relationship between hs-CRP and depression. Subgroup analysis was performed to investigate the combined effect of hs-CRP and each covariate on depression. Odds ratios (ORs) and 95% confidence intervals (CIs) were calculated to compare the prevalence of depression according to hs-CRP levels. All analyses were performed using SAS software, version 9.4 (SAS Institute, Cary, North Carolina, USA), and a P-value of <0.05 was considered significant.

## Electronic supplementary material

Supplementary tables

## Data Availability

This study analysed data from the 2016 KNHAES. All the KNHANES data are available to the public and can be downloaded from the KNHANES official website (http://knhanes.cdc.go.kr/).
