# Supplementary material for: Sex Difference in the Association between High-sensitivity C-reactive Protein and Depression: The 2016 Korea National Health and Nutrition Examination Survey
Source: Sci Rep. 2019 Feb 13;9:1918. doi: 10.1038/s41598-018-36402-3 (PMC6374396; doi:10.1038/s41598-018-36402-3)
Supplement: Supplementary file 1 — Supplementary tables [file 41598_2018_36402_MOESM1_ESM.docx]

**Sex Difference in the Association between High-sensitivity C-reactive Protein and Depression: The 2016 Korea National Health and Nutrition Examination Survey**

San Lee^1,4^, Sarah Soyeon Oh^1,2^, Sung-In Jang^1,3^, Eun-Cheol Park^1,3*^

1 Department of Public Health, Graduate School, Yonsei University, Seoul, Republic of Korea

2 Institute of Health Services Research, Yonsei University College of Medicine, Seoul, Republic of Korea

3 Department of Preventive Medicine, Yonsei University College of Medicine, Seoul, Republic of Korea

4 Department of Psychiatry and Institute of Behavioral Science in Medicine, Yonsei University College of Medicine, Seoul, Republic of Korea

*Corresponding author: [ecpark@yuhs.ac](mailto:ecpark@yuhs.ac)

| **Supplementary Table S1. Results of the multivariate linear regression analysis for the association between hs-CRP and Depression (PHQ-9 score)** | | | | | | |
| --- | --- | --- | --- | --- | --- | --- |
|  | **Depression Score (PHQ-9)** | | | | | |
|  | **Men** | | | **Women** | | |
|  | **β** | **SE** | **p-value** | **β** | **SE** | **p-value** |
| **Serum hs-CRP** | **0.067** | **0.026** | **0.009** | 0.041 | 0.033 | 0.223 |
| **Age (years)** |  |  |  |  |  |  |
| 20-29 | Ref. |  |  | Ref. |  |  |
| 30-39 | **0.889** | **0.300** | **0.003** | -0.192 | 0.363 | 0.598 |
| 40-49 | 0.481 | 0.316 | 0.128 | **-1.159** | **0.379** | **0.002** |
| 50-59 | 0.175 | 0.342 | 0.609 | **-1.984** | **0.485** | **<.0001** |
| 60-69 | -0.027 | 0.362 | 0.940 | **-2.409** | **0.544** | **<.0001** |
| 70-79 | -0.312 | 0.391 | 0.425 | **-2.418** | **0.575** | **<.0001** |
| ≥80 | -0.624 | 0.509 | 0.221 | **-3.341** | **0.665** | **<.0001** |
| **Educational attainment** |  |  |  |  |  |  |
| Elementary school and below | Ref. |  |  | Ref. |  |  |
| Middle school | -0.506 | 0.266 | 0.057 | 0.008 | 0.278 | 0.976 |
| High school | -0.366 | 0.230 | 0.112 | -0.432 | 0.259 | 0.096 |
| University or above | -0.328 | 0.242 | 0.175 | -0.552 | 0.286 | 0.054 |
| **Equalized household income** |  |  |  |  |  |  |
| Quartile 1 (low) | Ref. |  |  | Ref. |  |  |
| Quartile 2 | **-0.776** | **0.215** | **0.001** | **-1.150** | **0.233** | **<.0001** |
| Quartile 3 | **-1.175** | **0.221** | **<.0001** | **-1.240** | **0.244** | **<.0001** |
| Quartile 4 (high) | **-1.227** | **0.228** | **<.0001** | **-1.615** | **0.252** | **<.0001** |
| **Marital status** |  |  |  |  |  |  |
| Married | Ref. |  |  | Ref. |  |  |
| Separated/divorced/widowed | 1.533 | 0.272 | **<.0001** | 0.882 | 0.219 | **<.0001** |
| Never married | **1.173** | **0.242** | **<.0001** | 0.405 | 0.331 | 0.221 |
| **Alcohol use status** |  |  |  |  |  |  |
| No | Ref. |  |  | Ref. |  |  |
| Yes | -0.121 | 0.146 | 0.406 | 0.072 | 0.154 | 0.642 |
| **Smoking status** |  |  |  |  |  |  |
| Non-smoker | Ref. |  |  | Ref. |  |  |
| Smoker | 0.696 | 0.144 | **<.0001** | 2.598 | 0.377 | **<.0001** |
| **Chronic medical disease** |  |  |  |  |  |  |
| None | Ref. |  |  | Ref. |  |  |
| One | 0.264 | 0.180 | 0.143 | **0.766** | **0.216** | **0.001** |
| Two or more | **0.940** | **0.199** | **<.0001** | **1.091** | **0.241** | **<.0001** |
| **Residential area** |  |  |  |  |  |  |
| Urban | Ref. |  |  | Ref. |  |  |
| Rural | -0.008 | 0.133 | 0.952 | -0.027 | 0.146 | 0.852 |
| **BMI** |  |  |  |  |  |  |
| Underweight | **1.107** | **0.414** | **0.008** | 0.225 | 0.348 | 0.518 |
| Normal weight | Ref. |  |  | Ref. |  |  |
| Overweight | -0.041 | 0.139 | 0.767 | 0.057 | 0.171 | 0.739 |
| Obesity | **-0.670** | **0.294** | **0.018** | -0.618 | 0.330 | 0.061 |
| **Menopause (females only)** |  |  |  |  |  |  |
| No |  |  |  | Ref. |  |  |
| Yes |  |  |  | **0.679** | **0.347** | **0.050** |
| hs-CRP and PHQ-9 were analyzed as continuous variables. β, standardized regression coefficient, hs-CRP, high-sensitivity C-reactive protein; PHQ-9, patient health questionnaire-9; BMI, body mass index; SE, standard error | | | | | | |

| **Supplementary Table S2. Results of the multivariate logistic regression analysis for the association between hs-CRP and depression (PHQ-9 ≥10) in men stratified by chronic medical diseases** | | | | | | | | |
| --- | --- | --- | --- | --- | --- | --- | --- | --- |
|  | **Depression (PHQ-9≥10)** | | | | | | | |
|  | **Men without or with one  chronic medical disease** | | | | **Men with two or more  chronic medical diseases** | | | |
|  | **OR** | **95% CI** | | ***p*-value** | **OR** | **95% CI** | | ***p*-value** |
| **Serum hs-CRP** |  |  |  |  |  |  |  |  |
| Low: less than or equal to 3.0 mg/L | 1.00 |  |  |  | 1.00 |  |  |  |
| High: above 3.0 mg/L | **2.12** | **1.06** | **4.24** | **0.033** | 1.46 | 0.53 | 4.01 | 0.462 |
| **Educational attainment** |  |  |  |  |  |  |  |  |
| Elementary school and below | 1.00 |  |  |  | 1.00 |  |  |  |
| Middle school | **0.27** | **0.08** | **0.91** | **0.035** | 0.90 | 0.28 | 2.85 | 0.853 |
| High school | **0.29** | **0.11** | **0.73** | **0.009** | 1.05 | 0.36 | 3.02 | 0.936 |
| University or above | **0.29** | **0.11** | **0.76** | **0.012** | 0.47 | 0.11 | 2.08 | 0.321 |
| **Equalized household income** |  |  |  |  |  |  |  |  |
| Quartile 1 (low) | 1.00 |  |  |  | 1.00 |  |  |  |
| Quartile 2 | **0.44** | **0.22** | **0.87** | **0.019** | 0.49 | 0.18 | 1.35 | 0.167 |
| Quartile 3 | **0.24** | **0.11** | **0.52** | **<.0001** | **0.24** | **0.06** | **0.97** | **0.045** |
| Quartile 4 (high) | **0.20** | **0.09** | **0.46** | **<.0001** | 0.12 | 0.01 | 1.08 | 0.059 |
| **Marital status** |  |  |  |  |  |  |  |  |
| Married | 1.00 |  |  |  | 1.00 |  |  |  |
| Separated/divorced/widowed | **3.89** | **1.76** | **8.61** | **0.001** | **3.81** | **1.47** | **9.86** | **0.006** |
| Never married | **2.55** | **1.27** | **5.12** | **0.009** | 1.84 | 0.25 | 13.49 | 0.547 |
| **Smoking status** |  |  |  |  |  |  |  |  |
| Non-smoker | 1.00 |  |  |  | 1.00 |  |  |  |
| Smoker | **2.00** | **1.18** | **3.40** | **0.010** | **3.23** | **1.32** | **7.94** | **0.011** |
| **BMI** |  |  |  |  |  |  |  |  |
| Underweight | 1.94 | 0.65 | 5.83 | 0.238 | 1.62 | 0.20 | 13.16 | 0.651 |
| Normal weight | 1.00 |  |  |  | 1.00 |  |  |  |
| Overweight | 0.83 | 0.46 | 1.50 | 0.537 | 0.79 | 0.33 | 1.90 | 0.603 |
| Obesity | 1.11 | 0.40 | 3.06 | 0.839 | 0.25 | 0.03 | 2.27 | 0.216 |
| PHQ-9, patient health questionnaire-9; hs-CRP, high-sensitivity C-reactive protein; BMI, body mass index; OR, odds ratio; CI, confidence interval | | | | | | | | |
